# Developments in Binuclear α-Diimine Nickel Catalysts for Ethylene Polymerization

**DOI:** 10.3390/polym18161930

**Published:** 2026-08-07

**Authors:** Jingshuang Yang, Rong Gao, Qingqiang Gou, Randi Zhang, Tian Liu, Jingjing Lai, Ying Wang, Qiang Yue

**Affiliations:** Department of Polyethylene, SINOPEC (Beijing) Research Institute of Chemical Industry Co., Ltd., Beijing 100013, China; gaor.bjhy@sinopec.com (R.G.); gouqq.bjhy@sinopec.com (Q.G.); zhangrd.bjhy@sinopec.com (R.Z.); liutian.bjhy@sinopec.com (T.L.); laijj.bjhy@sinopec.com (J.L.); wangying.bjhy@sinopec.com (Y.W.); yueq.bjhy@sinopec.com (Q.Y.)

**Keywords:** binuclear liangd, α-diimine nickel catalyst, cooperative effect, chain-walking polymerization, polyethylene

## Abstract

Binuclear α-diimine nickel catalysts have attracted significant interest in ethylene polymerization due to their distinctive metal–metal cooperativity, which enables catalytic properties not achievable with mononuclear systems. This review summarizes the structural design principles and recent advances of these binuclear catalysts, with an emphasis on how different bridging architectures such as rigid aryl, flexible chain, N-heterocyclic, and specialty bridges regulate intermetallic distance, steric environment, and electronic effects. The impact of these structural factors on catalytic activity, polymer molecular weight, branching density, and mechanical properties is discussed. By establishing structure–performance relationships, this review provides a foundation for the rational design of binuclear nickel catalysts for advanced polyethylene synthesis.

## 1. Introduction

Polyolefins are the largest class of synthetic polymers worldwide by production volume and are widely used in plastics, elastomers, and fibers. Polyolefin synthesis can be categorized according to the underlying polymerization mechanisms into coordination polymerization [1,2,3], radical polymerization [4,5], ionic polymerization [6,7], metathesis polymerization [8,9], and emerging approaches such as photo- and electrochemically triggered polymerization [10,11]. Each method exhibits distinct advantages and limitations. On an industrial scale, coordination and radical polymerization continue to dominate the production of bulk polyolefin materials. Between the two, coordination polymerization is particularly advantageous owing to its ability to afford controlled polymer microstructures under relatively mild conditions.

As the most representative variety of polyolefin materials, polyethylene (PE) has found its applications permeating virtually every facet of modern life, ranging from everyday commodity items such as food packaging films and lightweight shopping bags, to load-bearing hollow containers, pressure-resistant and corrosion-resistant municipal water supply and drainage pipes and further extending to wire and cable insulation layers that demand stringent material performance, as well as load-bearing components in artificial joints within the biomedical field. Owing to the tunability of its molecular weight and crystallinity, polyethylene is commercially available in a series of grades, including low-density polyethylene (LDPE), high-density polyethylene (HDPE), and ultra-high-molecular-weight polyethylene (UHMWPE), which collectively enable its versatile deployment across a broad spectrum of requirements, from flexible packaging to rigid structural applications and from routine consumer uses to advanced medical devices. Such remarkable adaptability has established polyethylene as an indispensable foundational material supporting both modern industry and public welfare.

Catalyst molecular design is central to achieving structural diversity and performance customization of polyolefins [1,3,12,13,14,15,16]. Since Brookhart’s 1995 seminal report on α-diimine nickel/palladium catalysts, late-transition metal nickel systems have become a cornerstone of polyolefin catalysis research, owing to their unique chain-walking mechanism [17,18,19,20,21,22,23,24,25,26,27,28,29,30]. Nonetheless, conventional mononuclear catalysts remain challenged by inadequate thermal stability and limited precision in regulating polymer microstructures, including branching density and molecular weight distribution (MWD).

**Figure 1:**
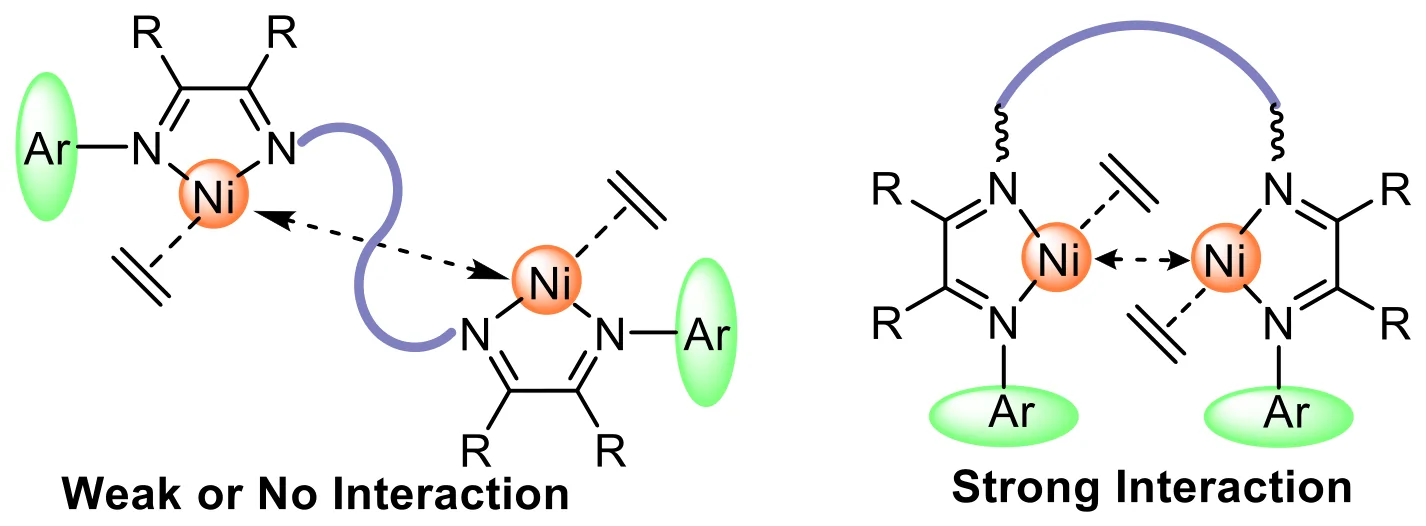
Structural scheme of a binuclear α-diimine nickel catalyst.

To overcome the performance limitations inherent to mononuclear catalysts, researchers have drawn inspiration from the catalytic mechanisms of metalloenzymes [31,32,33]. In many such enzymes, two or more metal centers are positioned in close proximity and function cooperatively to simultaneously activate electrophilic and nucleophilic substrates, thereby achieving remarkable catalytic activity and selectivity. This cooperative activation mode of multi-metal centers has directed research efforts toward the development of binuclear and even multinuclear homogeneous catalytic systems [34,35,36,37,38,39,40,41,42]. Studies have demonstrated that synergistic reactivity patterns involving two or more metal centers are strongly dependent on the catalyst architecture [43,44,45,46,47]. Significant metal–metal cooperativity is observed when the two active centers are appropriately connected through a bridging group to establish well-designed interactions. Conversely, in the absence of such interactions, the cooperative effect is unlikely to be manifested (Figure 1).

In binuclear α-diimine nickel catalytic systems, the design of the bridging structure plays a pivotal role in modulating metal–metal cooperativity. The distance between the two active centers and the nature of the substituents have been identified as two core parameters influencing the cooperative effects between the metal centers in olefin polymerization. Furthermore, the overall geometry of the multinuclear catalyst profoundly impacts its catalytic performance. The chemical nature and geometric characteristics of the bridging unit directly determine the coordination environment and spatial orientation of the bimetallic centers, thereby profoundly influencing catalytic activity, thermal stability, and polymer microstructure through the regulation of metal–metal interactions. Based on the chemical nature and geometric features of the bridges, binuclear α-diimine nickel catalytic systems can be classified into distinct categories.

Based on this review, binuclear α-diimine nickel catalytic systems are classified into four major categories: rigid aryl-bridged, flexible chain-bridged, N-heterocyclic-bridged, and special unsymmetric catalysts. The following sections systematically review the molecular design strategies and structural characteristics of these four catalyst classes, as well as their regulatory effects on catalytic behavior and polymer properties in ethylene polymerization. This review aims to provide theoretical guidance for the design and development of high-performance binuclear catalysts and to offer perspectives on future directions in this research field.

## 2. Rigid Aryl-Bridged Catalysts

In the field of bridging architectures, the rigid aryl-bridged type has been the subject of the most extensive research to date. Depending on the degree of conjugation, spatial configuration, and electronic effects of the aromatic ring system, binuclear metal catalysts can be further subdivided into the following four subtypes: mono-benzene-bridged catalysts, bulky *o*-phenylene-bridged catalysts, fused-ring aromatic-bridged catalysts, and methylene-containing diaryl-bridged catalysts. This structural classification facilitates understanding of structure–activity relationships in catalysis and provides an important theoretical basis for the targeted design and synthesis of binuclear nickel systems with specific catalytic performance.

### 2.1. Mono-Benzene-Bridged Catalysts

Mono-benzene-bridged binuclear complexes refer to a class of compounds in which the two metal centers are connected through a single aryl framework linking the imine bonds. In recent years, research groups such as that of Mostafa Khoshsefat have conducted a series of studies on this type of structure, systematically investigating their synthetic methods, catalytic performance, and polymer structural characteristics [48,49,50,51,52,53,54].

**Figure 2:**
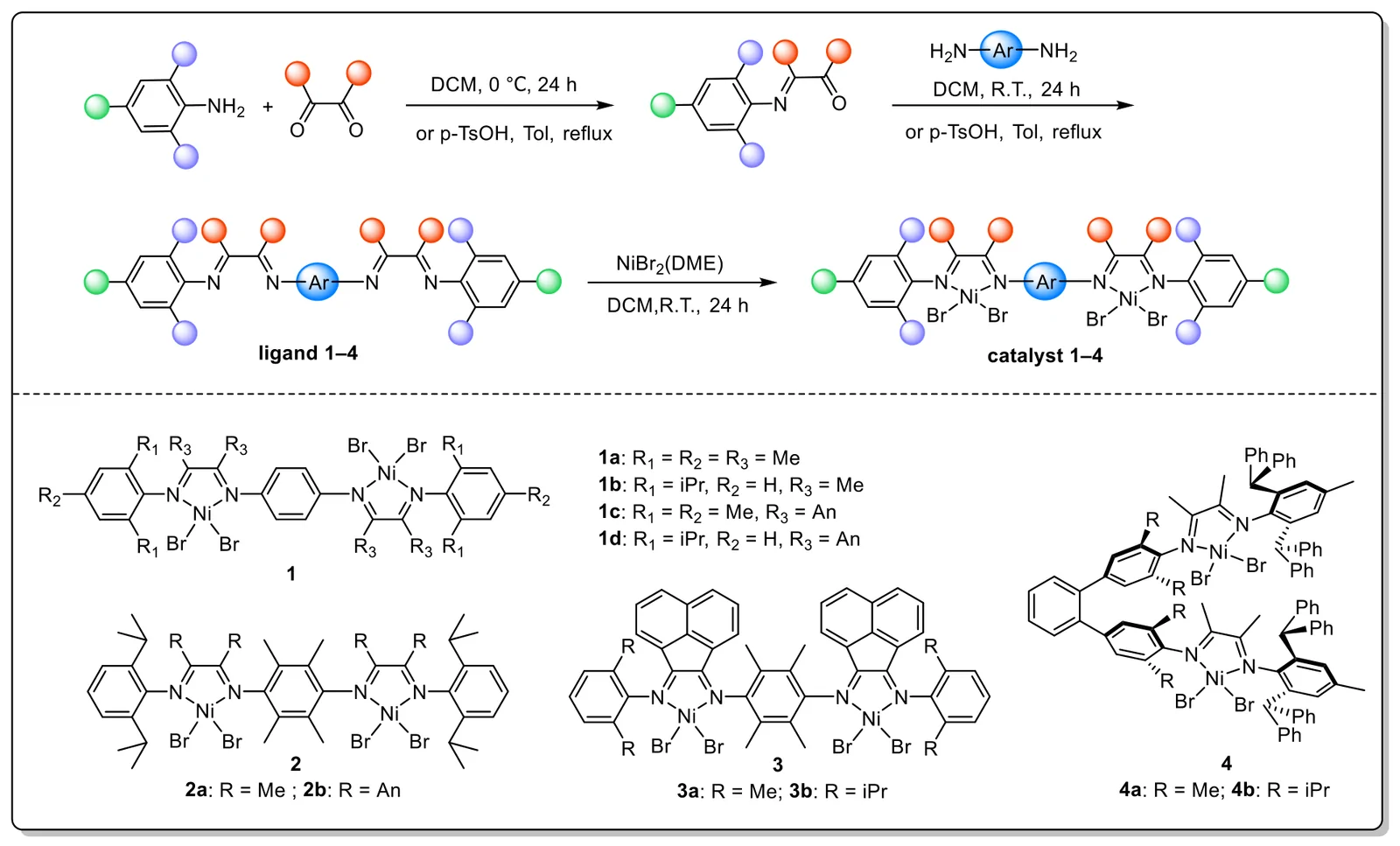
Synthesis and structural scheme of the mono-benzene-/bulky *o*-phenylene-bridged binuclear α-dimine nickel catalysts.

In 2016, Khoshsefat et al. [48] designed and synthesized a novel class of binuclear α-diimine Ni(II)-based catalysts (Figure 2 (**1**)) for ethylene polymerization. The ensuing results demonstrated that under conditions of [Al]/[Ni] = 2000/1 and *T*_p_ = 42 °C, catalyst **1d** exhibited a catalytic activity of 1.07 × 10^6^ g mol^−1^ h^−1^ when R_1_ = isopropyl (*i*Pr), R_2_ = H, and R_3_ = acenaphthylene (An). Theoretical analysis indicated that the steric and electronic effects of both the substituents and the diimine backbone exerted a significant influence on the catalyst behavior, such as catalytic activity, the viscosity average molecular weight and the degree of branching of the polymer products. Furthermore, GPC and DSC analyses revealed that, owing to the cooperative effect between the active centers, the polyethylene obtained from catalyst **1d** displayed a broad molecular weight distribution, and its crystallization melting curve exhibited the coexistence of a peak along with a wide shoulder. Increased ethylene pressure led to a higher polyethylene yield, as well as improved uniformity and regularity of chain growth. These effects were evidenced by changes in the DSC thermogram and corroborated by the greater morphological uniformity visible in SEM images.

In 2021, Zohuri et al. [49] further applied binuclear Ni-based catalyst **1d** to the copolymerization of methyl methacrylate (MMA) with 1-hexene, successfully obtaining copolymers with an MMA incorporation rate in the main chain as high as 96%. In this work, catalyst **1d** was appropriately employed to generate novel semiconducting materials with high resistance, which possesses a vacancy (hole) in its structure. The copolymer synthesized with equimolar fractions of the comonomers exhibited efficient electrical resistance without degradation and demonstrated good semiconducting properties.

Khoshsefat et al. [50] replaced the phenyl bridging with a tetramethylphenyl unit to design and synthesize a binuclear α-diimine nickel catalyst (Figure 2 (**2**)), which was employed in the polymerization of 1-hexene. Using ethylaluminum sesquichloride (EASC) as the efficient cocatalyst, the effects of various polymerization conditions on catalytic performance were systematically investigated. Compared with the mononuclear analog, the binuclear catalyst exhibited higher catalytic activity, and the resulting polymer also showed a significantly increased viscosity average molecular weight. Notably, the synergistic effect of high local monomer concentration, steric hindrance, and electronic effects accounts for the pronounced chain walking of catalyst **2b**. In addition, longer α-olefins (1-octene and 1-decene) enhanced productivity, conversion, molecular weight, and narrowed MWD, with lower branching density. Elevated temperatures further confirmed the superior thermal stability of binuclear over mononuclear catalysts.

In 2022, Mortazavi et al. [51] employed modified methylaluminoxane (MMAO) as a cocatalyst and selected the binuclear α-diimine nickel catalyst **2b** to elucidate the homopolymerization mechanism of 5-ethylidene-2-norbornene (ENB). The findings indicated that the maximum polymerization activity of the binuclear catalyst was considerably higher than that of its mononuclear analog. In addition, the catalyst structure was identified as a primary factor influencing the glass transition temperature (*T*_g_) of the resulting cyclic polyolefins. The polymer obtained from the binuclear catalyst demonstrated a *T*_g_ of 176 °C, while the polymer derived from the mononuclear catalyst exhibited a *T*_g_ of 140 °C. Dynamic mechanical thermal analysis (DMTA) further confirmed that variations in catalyst structure exert a profound influence on the microstructure of the polymer chains.

The same group [52] expanded upon their previous research, continuing to utilize MMAO as the cocatalyst. They conducted a systematic investigation into the catalytic behavior of the binuclear α-diimine nickel catalyst **2b** in the copolymerisation of ENB and norbornene (NB). This investigation resulted in the successful preparation of a series of copolymers with varying ENB molar ratios. The study found that catalytic activity tended to increase with a higher proportion of ENB in the feed, and the binuclear catalyst consistently exhibited superior activity relative to the mononuclear catalyst throughout the copolymerization process. It is noteworthy that beyond addition polymerization, a detailed analysis of the polymer microstructures ^1^HNMR unexpectedly indicated the concomitant occurrence of cationic and ring-opening polymerization pathways during copolymerization, though their respective contributions differed significantly.

Zohuri et al. [53] synthesized a series of binuclear α-diimine nickel catalysts featuring an anthraquinone backbone by varying the ortho-substituents of the aniline moiety (Figure 2 (**3**)) and applied them in the synthesis of polynorbornene. The findings demonstrated that the binuclear catalysts exhibited superior catalytic efficiency, and the resulting polynorbornenes generally possessed elevated molecular weights (*M*_w_) in comparison to those obtained with mononuclear catalysts. Thermal analyses, incorporating DSC, DMTA and TGA, further revealed that the polymer produced by catalyst **3b** possessed a more uniform microstructure and higher stereoregularity.

Khoshsefat et al. [54] employed the binuclear nickel catalyst **3b** for the homopolymerization and copolymerization of 1-hexene with MMA, using MMAO as the cocatalyst. The findings demonstrated that, in comparison to the mononuclear analog, the binuclear catalyst **3b** exhibited significant advantages in terms of both catalytic activity and copolymer molecular weight. Remarkably, the addition of MMA (up to [MMA]/[1-hexene] = 50/50 molar ratio) boosted the catalytic activity across all catalysts. ^1^HNMR analysis further established a clear correlation between increased MMA feed composition and enhanced comonomer incorporation, a trend quantitatively supported by reactivity ratios derived from the Kelen–Tudos method.

### 2.2. Bulky o-Phenylene-Bridged Catalysts

In mono-benzene-bridged structures, the two metal centers are connected via a single aryl unit, rendering their spatial distance relatively fixed. The cooperative effects in such systems rely primarily on the steric hindrance imparted by the ortho-substituents of the aniline and the electronic effect of the ligand backbone. To further expand the tunability of the intermetallic distance and to investigate the impact of bridge rigidity and conjugation degree on catalytic behavior, researchers have extended the bridging unit from a single benzene ring to a bulky *o*-phenylene-bridged structure. Only a limited number of bimetallic α-diimine catalysts featuring sterically bulky substituents on *o*-aryl positions have been documented to date.

Motivated by the established bimetallic synergistic effects in epoxide/CO_2_ copolymerization [55], Liu et al. [56] proceeded to design and synthesize a series of bulky *o*-phenylene-bridged bimetallic α-diimine Ni(II) catalysts (Figure 2 (**4**)) and systematically investigate their catalytic performance in ethylene polymerization. The findings demonstrated that, in comparison to their mononuclear analogs, the binuclear Ni(II) catalysts **4** exhibited elevated catalytic activity (up to 4.80 × 10^6^ g mol^−1^ h^−1^). Concurrently, the resultant polymers were characterized by elevated molecular weights and diminished branching densities.

Furthermore, by modulating the catalyst structure and polymerization conditions, all prepared polyethylenes displayed elastomeric behavior, with their mechanical properties tunable over a wide range. It is noteworthy that the polyethylene material produced by catalyst **4b** achieved a strain recovery rate (SR) of up to 86%, a value comparable to the optimal recovery rates reported for polyethylene thermoplastic elastomers, thereby demonstrating outstanding elastic performance.

### 2.3. Fused-Ring Aromatic-Bridged Catalysts

Beyond simple mono-benzene and *o*-phenylene moieties, fused-ring aromatic system serve as widely adopted bridging units in the construction of binuclear α-diimine catalysts, a utility derived from their unique conjugated nature and geometric conformation. These bridging structures provide a means to regulate both the spatial separation and mutual alignment of the bimetallic centers, while also potentially tuning catalytic performance via electronic modulation. Consequently, they offer a promising molecular design approach for the development of advanced polyolefin materials.

**Figure 3:**
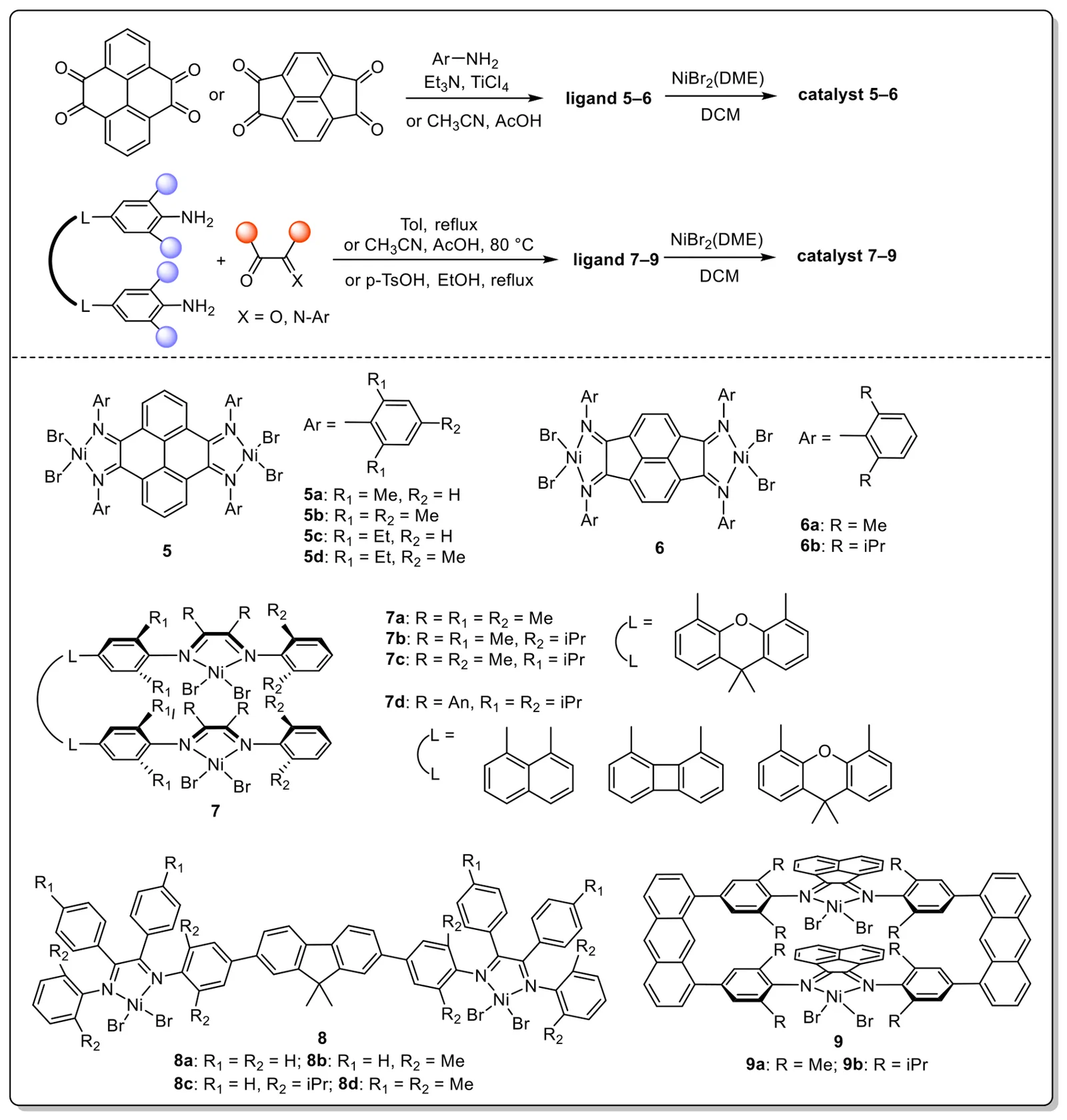
Synthesis and structural scheme of the fused-ring aromatic-bridged binuclear α-dimine nickel catalysts.

Sun et al. [57] synthesized a novel class of binuclear α-diimine nickel complexes (Figure 3 (**5**)) bearing 4,5,9,10-tetra(arylimino)-pyrenylidenes based on the planar conjugated pyrene-4,5,9,10-tetraone backbone via condensation with a series of aniline derivatives. A comprehensive characterization of the ligands and complexes was conducted, and their bimetallic molecular structures were confirmed by single-crystal X-ray diffraction. The study demonstrated that, when employing methyl aluminoxane (MAO) or dimethylaluminum chloride (Me_2_AlCl) as cocatalysts, this series of binuclear nickel catalysts exhibited high catalytic activity in ethylene polymerization along with excellent prolonged period, maintaining an activity of up to 1.30 × 10^6^ g(PE) mol(Ni)^−1^ h^−1^.

Fu et al. [58] designed and synthesized a series of α-diimine binuclear nickel catalysts with a conjugated backbone (Figure 3 (**6**)). A systematic investigation was conducted to ascertain the influence of catalyst structure, cocatalyst ratio, polymerization time, and solvent on the catalytic activity, polymer molecular weight, and branching architecture. The experiments demonstrated that, upon activation with MMAO, these binuclear nickel catalysts efficiently mediated ethylene polymerization, achieving a maximum activity of 1.05 × 10^6^ g(PE) mol(Ni)^−1^ h^−1^. Notably, under identical polymerization conditions, the activity of catalyst **6b** was twice that of its corresponding mononuclear analog, underscoring the promotional effect of the binuclear architecture on catalytic performance.

In 2021, the same group [59] further explored the application of catalyst **6b** in ethylene polymerization using alkyl aluminum and organoboron compounds as binary cocatalyst system. The study revealed a close correlation between the enhancement in catalytic activity and the nature of the organoboron compound, the type of alkyl aluminum, and the catalyst structure. When combined with diethylaluminum chloride (Et_2_AlCl), the introduction of sodium tetrakis [3,5-bis(trifluoromethyl)phenyl]borate (NaBArF) significantly promoted ethylene polymerization at elevated temperatures, with a 41.3% increase in activity observed at 70 °C. This binary cocatalyst system has been demonstrated to reduce the required molar ratio of [Al]/[Ni], while concomitantly increasing the number of active centers and prolonging their lifetime. These effects offer a viable pathway for industrial ethylene polymerization under high-temperature conditions.

Chen et al. [60] reported a class of dinuclear α-diimine Ni(II) catalysts featuring a xanthene-bridged scaffold (Figure 3 (**7a**–**7c**)), detailing their synthesis, structural characterization, and ethylene polymerization behavior of these catalysts. The desired dianiline utilized in this series of ligands can be readily obtained on a multigram scale without using column chromatography. The results of the polymerization demonstrated that these catalysts exhibited both good thermal stability and high polymerization activity, with the maximum activity reaching 5.00 × 10^6^ g(PE) mol(Ni)^−1^ h^−1^. Notably, they retained high activity exceeding 10^6^ g(PE) mol(Ni)^−1^ h^−1^ even at an elevated temperature of 80 °C. The resulting polyethylenes were characterized by high molecular weight, narrow MWD, and exceptionally low branching density, which the researchers attributed to a metal–metal cooperativity effect between the two nickel centers that effectively suppresses *β*-H elimination and chain walking.

To further expand the structural diversity of the bridging, the Chen group [61] introduced naphthalene- and biphenylene-bridged moieties, synthesizing a new series of binuclear α-diimine Ni(II) catalysts (Figure 3 (**7d**)). In comparison to their mononuclear counterparts, these binuclear catalysts demonstrated enhanced activity, higher molecular weight, and significantly reduced branching density in the process of ethylene polymerization. DFT calculations revealed that the Ni-Ni distances across the different bridging scaffolds (naphthalene-, biphenylene-, and xanthene-bridged structures) were comparable, thereby accounting for the largely consistent catalytic performance observed across the series.

Yang et al. [62] synthesized four fluorene-based binuclear α-diimine Ni catalysts (Figure 3 (**8**)) and systematically evaluated the influence of catalyst structure on polymerization activity, polymer branching density, and molecular weight using Et_2_AlCl as the cocatalyst. This work demonstrated that the selection of ligand structure is paramount in the modulation of the microstructure of the resultant polymer. In this study, the effects of catalyst concentration, the molar ratio of [Al]/[Ni], and the ethylene pressure on polymerization behavior were investigated using catalyst **8d** as a representative selection. The findings indicated that the polymerization activity attained a maximum of 12,437 g(PE) g(cat)^−1^, suggesting a promising prospect for potential practical applications.

Jian et al. [63] ingeniously integrated the rigid constraint of a macrocyclic framework with the cooperative effect of bimetallic centers into the classical α-diimine nickel catalytic system, designing and synthesizing a class of structurally unique macrocyclic binuclear α-diimine nickel catalysts (Figure 3 (**9**)). A series of studies have demonstrated that, in comparison with classical Brookhart-type acyclic mononuclear α-diimine nickel catalysts, this innovative binuclear catalyst demonstrates higher thermal stability (up to 110 °C) in the context of MAO-activated ethylene polymerization. Furthermore, the production of polyethylene is observed to be accompanied by a substantial augmentation in molecular weight, alongside a considerable reduction in branching density (9/1000C). It is noteworthy that by merely adjusting the ortho-aryl substituents, a bimodal polyethylene with a broad molecular weight distribution (MWD = 8.08~14.66) could be synthesized using a single catalyst. This finding provides a novel approach for the precise synthesis of diverse polyethylene materials.

### 2.4. Methylene-Containing Diaryl-Bridged Catalysts

A class of methylene-containing diaryl-based multinuclear α-diimine nickel complexes can be synthesized via the condensation of a diketone backbone with methylene-bridged dianiline and aniline derivatives bearing various substituents. By modulating the intermetallic distances between bimetallic or multimetallic centers through the bridging moiety, this type of catalysts provides an effective molecular design strategy for investigating metal–metal cooperative effects and optimizing polymerization performance.

**Figure 4:**
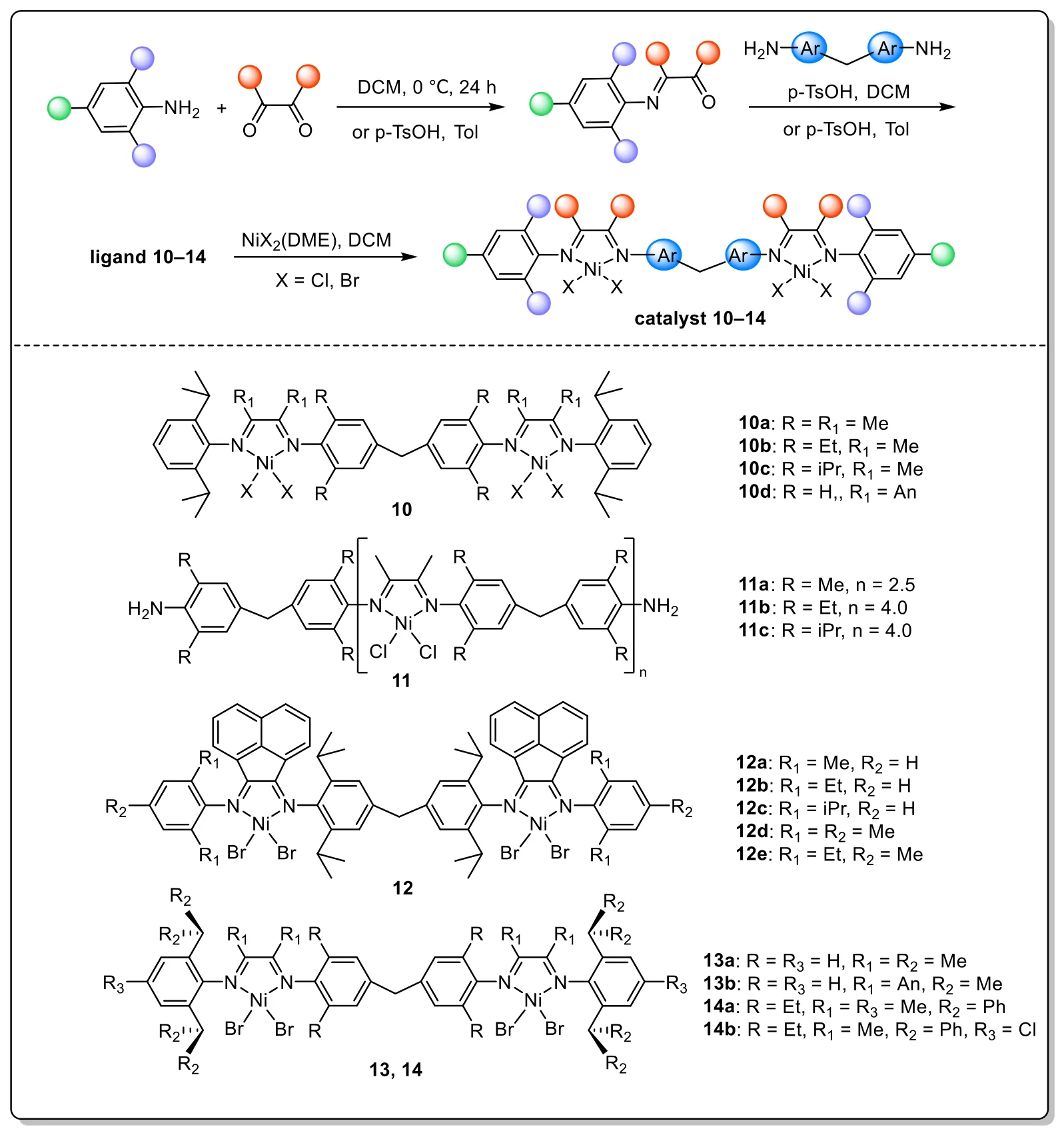
Synthesis and structural scheme of the methylene-containing diaryl-bridged multinuclear α-dimine nickel catalysts.

In 2004, Schumann et al. [64] pioneered the preparation of a series of structurally well-defined binuclear nickel complexes (Figure 4 (**10**)) via Schiff base condensation of 2,3-butanedione, 2,6-diisopropylaniline, and substituted methylene-bridged bis-aniline. Structural analysis revealed that, compared to the mono-nickel-center catalysts, the molecules of the new catalysts were significantly bigger, and the intermetallic distance between the two active centers was effectively controlled, thereby enabling precise modulation of the microchemical environment around each nickel center. Ethylene polymerization evaluation demonstrated that the binuclear architecture exerts a pronounced influence on catalytic activity when the substituent R was isopropyl or ethyl (Figure 4 (**10b**,**10c**)), the activities of the binuclear catalysts substantially exceeded those of the corresponding mononuclear analogs. In contrast, when the substitute was methyl (Figure 4 (**10a**)), the new catalyst exhibited lower activity than its mononuclear reference.

The group further prepared multinuclear nickel catalysts (Figure 4 (**11**)) through direct condensation of 2,3-butanedione with substituted methylene-bridged bis-aniline, thereby extending the scope of spatial control over multiple active centers. Among these, the multinuclear catalyst bearing ethyl substituents (Figure 4 (**11b**)) displayed optimal catalytic performance at 25 °C with an [Al]/[Ni] ratio at 500, achieving an activity of 3220 g(PE) g(Ni)^−1^ h^−1^.

In 2013, Sun et al. [65] synthesized a series of structurally well-defined α-diimine binuclear nickel complexes (Figure 4 (**12**)) using isopropyl-substituted dianiline as a key intermediate, and their molecular structures were confirmed through single-crystal X-ray diffraction. Upon activation with Et_2_AlCl or MAO, these catalysts exhibited exceptionally high ethylene polymerization activities on the order of 10^6^ g(PE) mol(Ni)^−1^ h^−1^ over a temperature range from room temperature to 50 °C. Notably, catalyst **12d** achieved a maximum activity of 1.59 × 10^7^ g(PE) mol(Ni)^−1^ h^−1^. Compared with mononuclear nickel analogs, the binuclear complexes not only displayed higher activities but also yielded polyethylenes with simultaneously enhanced molecular weights and branching densities. This concurrent optimization of activity, molecular weight, and branching degree highlights the distinct advantages conferred by the binuclear architecture.

Khoshsefat et al. [66,67] synthesized binuclear α-diimine nickel catalysts (Figure 4 (**13**)) by varying the diketone backbone and systematically compared the influence of different bridging structures on catalytic performance. Further investigation revealed that, beyond the ligand backbone, the presence of a second metal center and its cooperative interplay with the bridging moiety are critical factors governing catalyst behavior. By tuning the nature and length of the bridging group, the electron density at the metal centers, catalyst stability, and polymerization performance can be effectively modulated. For instance, substituents with greater steric bulk confer higher electron density, thereby imparting enhanced stability and activity to the binuclear catalysts. The study identified that the catalyst possessing optimal steric hindrance exhibited the highest activity among all binuclear and mononuclear counterparts, reaching 1.6 × 10^6^ g(PE) mol(Ni)^−1^ h^−1^, along with the lowest short-chain branching density (32.5/1000C).

Dai et al. [68] designed and synthesized a class of methylene-bridged binuclear bulky dibenzhydryl α-diimine Ni(II) catalysts (Figure 4 (**14**)) and applied them to ethylene polymerization. These binuclear complexes exhibited high activity on the order of 10^6^ g(PE) mol(Ni)^−1^ h^−1^, and successfully produced highly branched polyethylenes (79~92/1000C) with high molecular weights (*M*_n_ up to 44.57 × 10^4^ g mol^−1^). The resulting polymeric materials demonstrated outstanding elastic recovery performance, with a SR value as high as 84%. Compared with mononuclear α-diimine nickel catalysts, these binuclear catalysts not only exhibited enhanced thermal stability and polymerization activity but also yielded polyethylenes with significantly higher molecular weights, thereby further substantiating the potential of binuclear cooperative effects in advancing the performance of polyolefin materials.

## 3. Flexible Chain-Bridged Catalysts

In the design of binuclear α-diimine nickel catalysts, beyond the extensively investigated rigid aryl-bridged architectures, the introduction of flexible chain as a bridging unit between the two metal centers represents an equally intuitive and effective design strategy. However, studies on flexible chain-bridged binuclear nickel catalysts remain comparatively limited in the extant literature when compared to their rigid-bridged counterparts. This class of catalysts enables precise modulation of both the intermetallic distance and the relative orientation of the two metal centers simply by varying the number of methylene (-CH_2_-) units within the flexible tether, thereby exerting direct control over the resultant catalytic behavior.

**Figure 5:**
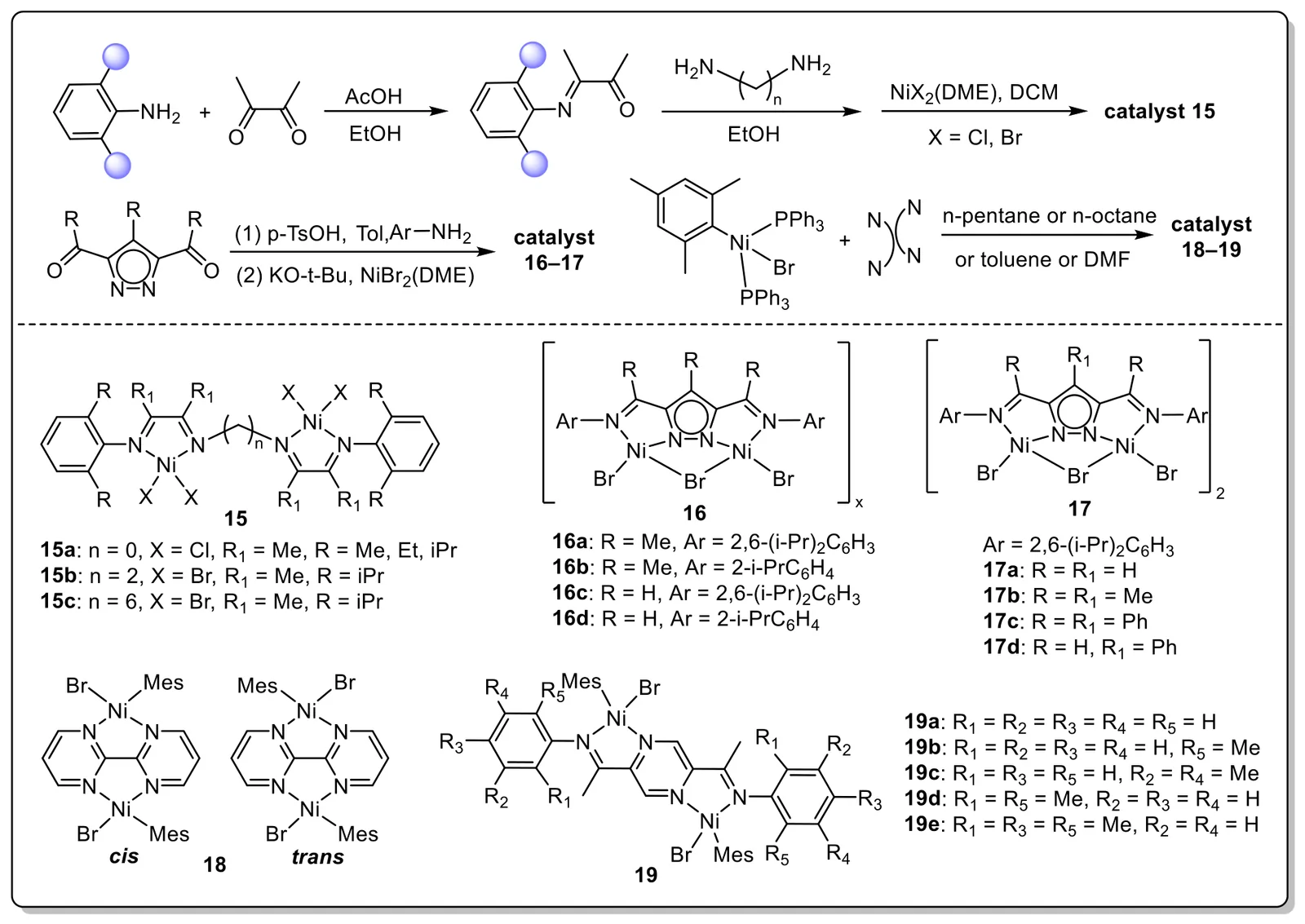
Synthesis and structural scheme of the flexible chain-/N-heterocyclic-bridged binuclear α-dimine nickel catalysts.

Revankar et al. [69] provided a versatile set of binuclear α-diimine nickel complexes (Figure 5 (**15a**)) featuring two nickel centers connected via a direct bond. In combination with MAO as a cocatalyst, these catalysts efficiently mediate ethylene oligomerization. At 30 °C and an [Al]/[Ni] ratio of 300/1, the catalytic system predominantly produced oligomeric fractions C2~C20, achieving a catalytic activity as high as 1123 kg(oligomer) mol(Ni)^−1^ bar^−1^. Substituent-induced shifts in metal center potential, verified by cyclic voltammetry, correlated strongly with oligomerization activity. Trace polyethylene formation occurred with C1 and C3 at 30 °C, but selectivity shifted entirely to oligomers at higher temperatures for all complexes.

Khoshsefat et al. [66,67,70] further extended the flexible bridge strategy by introducing ethyl and hexyl spacers between the two metal centers, thereby designing and synthesizing binuclear α-diimine nickel catalysts **15b** (ethyl-bridged) and **15c** (hexyl-bridged) with distinct flexible tether lengths (Figure 5). Their catalytic performance was systematically investigated in both 1-hexene homopolymerization and ethylene homopolymerization. The experimental revealed that, in 1-hexene polymerization, the mononuclear catalyst proceeded via a conventional insertion pathway, predominantly yielding poly(1-hexene) containing a high density of butyl branches. In contrast, the binuclear catalysts **15b** and **15c**, influenced by a partial chain-walking mechanism, produced poly(1-hexene) with a notably higher content of less common short-chain branches, such as ethyl and propyl branches. Computational investigation of the dinuclear catalysts corroborated the presence of different stereoisomers (syn and anti), linking their existence to the broadened MWD, the altered polymerization pathway, and the unique short-chain branching (SCB) curves.

In ethylene homopolymerization, the mononuclear catalyst yielded highly branched, amorphous polyethylene. In contrast, the polyethylenes produced by the binuclear catalysts **15b** and **15c** exhibited pronounced methyl selectivity in their branching architecture. The polymer chains derived from these binuclear catalysts developed crystalline sequences with high melting points (*T*_m_), and their melting temperatures even exceeded that of ideally 100% crystalline polyethylene, thereby displaying chain structural characteristics reminiscent of ethylene/propylene copolymers analogs.

More importantly, this work systematically compared the influence of different bridging architectures (catalysts **1a**, **2**, **10d**, **15b**, and **15c**) on polymerization performance. The pronounced hexyl branch selectivity of catalyst **2b** evidenced cooperative bimetallic interactions, whereas the methyl branch preference of catalysts **15b** and **15c** produced crystalline domains with higher melting points. The elevated vinyl end-group content observed for catalyst **10d** correlated with the larger intermetallic Ni···Ni distance.

Kinetic profiling and computational analysis of the precatalysts and catalysts established that the polymerization rate constant correlates directly with both the precatalyst–catalyst energy gap and the Et···methyl cationic active center interaction energy. A small energy gap (low activation barrier) ensures rapid activation (mononuclear nickel catalyst), whereas strong ethylene binding drives high monomer uptake and productivity (catalyst **2b**). Accordingly, theoretical parameters including electron affinity, Ni Mulliken charge, chemical potential and hardness, and global electrophilicity indicated catalyst **2b** as the optimal system.

## 4. N-Heterocyclic Bridged Catalysts

Beyond the aforementioned bridging strategies, nitrogen-containing heterocycles have also been employed to construct structurally novel binuclear or multinuclear olefin polymerization catalysts, owing to their versatile coordination modes and potential electron-mediating capabilities.

Meyer et al. [71,72,73,74] synthesized a series of pyrazolate-based dinuclear α-diimine ligands bearing different backbone substituents and steric hindrance. Subsequent to the reaction of these ligands with NiBr_2_(DME), mass spectrometry analysis indicated the formation of an oligonuclear species [LNi_2_Br_3_]_x_ (Figure 5 (**16**)). This conclusion was confirmed through single-crystal X-ray diffraction structure of [LNi_2_Br_3_]_3_. The binuclear ligand framework effectively prevented the dissociation of the complex into mononuclear species and maintained the adjacent nickel centers in suitable spatial orientation. This may have enabled cooperative effects during ethylene polymerization. Structure–activity correlations demonstrated that substituents on the pyrazole backbone and bulky ortho-substituents on the aniline moiety were beneficial for ethylene polymerization activity. However, despite their unique structure, the polymerization activity of this novel class of pyrazole-imine binuclear nickel catalysts, as well as the microstructures (e.g., branching degree, *M*_w_, and *T*_m_) of the resulting polyethylene, did not exhibit significant differences compared to those obtained with conventional mononuclear cationic α-diimine nickel catalysts.

In 2008, the same group [75] further expanded the ligand library by introducing different backbone at the pyrazole-C^4^ and substituents such as -H, -Me, and -Ph of the imine, respectively, synthesizing a series of pyrazolate-bridged binuclear ligands with finely varied structural differences. Similar to the synthesis of catalyst **16**, the resulting nickel complexes exhibited a tetranuclear structure [L_2_Ni_4_Br_6_] (Figure 5 (**17**)), which contains a nearly planar rectangular arrangement of Ni_4_ with an unusual central μ_4_-bromide. When this multinuclear nickel catalysts was applied to the addition polymerization of norbornene, it was found that their catalytic activity was highly sensitive to solvent. The resulting polynorbornenes (PNB) were noncrystalline and insoluble in conventional solvents, but exhibited a *T*_g_.

Klein et al. [76] designed and synthesized a bipyrimidine-bridged binuclear nickel complex (Figure 5 (**18**), Mes = 2,4,6-trimethylphenyl), and thoroughly characterized it using electrochemical and various spectroscopic techniques. The long-wavelength absorption features of the binuclear complex indicated a significant degree of electronic coupling between the two nickel centers through the low-lying unoccupied π* orbital (LUMO) of the bridging ligand. Electrochemical studies revealed that while the mononuclear analog underwent rapid bromide dissociation upon one-electron reduction, the binuclear nickel complex **18** exhibited stable, reversible reduction electrochemistry and generated radical anion species with spin density predominantly localized on the ligand center. Structural analysis revealed that the mononuclear complex exhibited shorter Ni-N bonds, thereby favoring optimal orbital overlap. Conversely, in the binuclear complex **18**, the bulky [Ni(Mes)Br] fragments impeded the concurrent attainment of perfect adaptation to both chelating sites of the bipyrimidine bridge, thereby partially weakening the orbital overlap between the metal and the ligand.

In 2012, the Klein’s group [77] further extended the bridging unit to pyrazine, preparing and characterizing the bis(arylimino)-1,4-pyrazine binuclear nickel complex (Figure 5 (**19**)). The researchers innovatively combined nuclear magnetic resonance (NMR) spectroscopy with quantum chemical calculations to successfully assign the stereoisomers of the complex and determine their relative stability. Analogous to complex **18**, the two nickel centers in complex **19** also exhibited substantial electronic coupling through the low-lying π* orbital of the bridging ligand. This complex underwent a reversible reduction process, resulting in the formation of a stable anionic complex. The spin density of this complex was found to be localized on the ligand-centered radical.

## 5. Special Unsymmetric Catalysts

In recent years, in addition to classical structures, binuclear nickel catalysts with special unsymmetric molecular architectures including homo/hetero-nuclear dimetallic have also been successively developed, providing new research avenues for the structural regulation and performance expansion of polyolefin materials.

**Figure 6:**
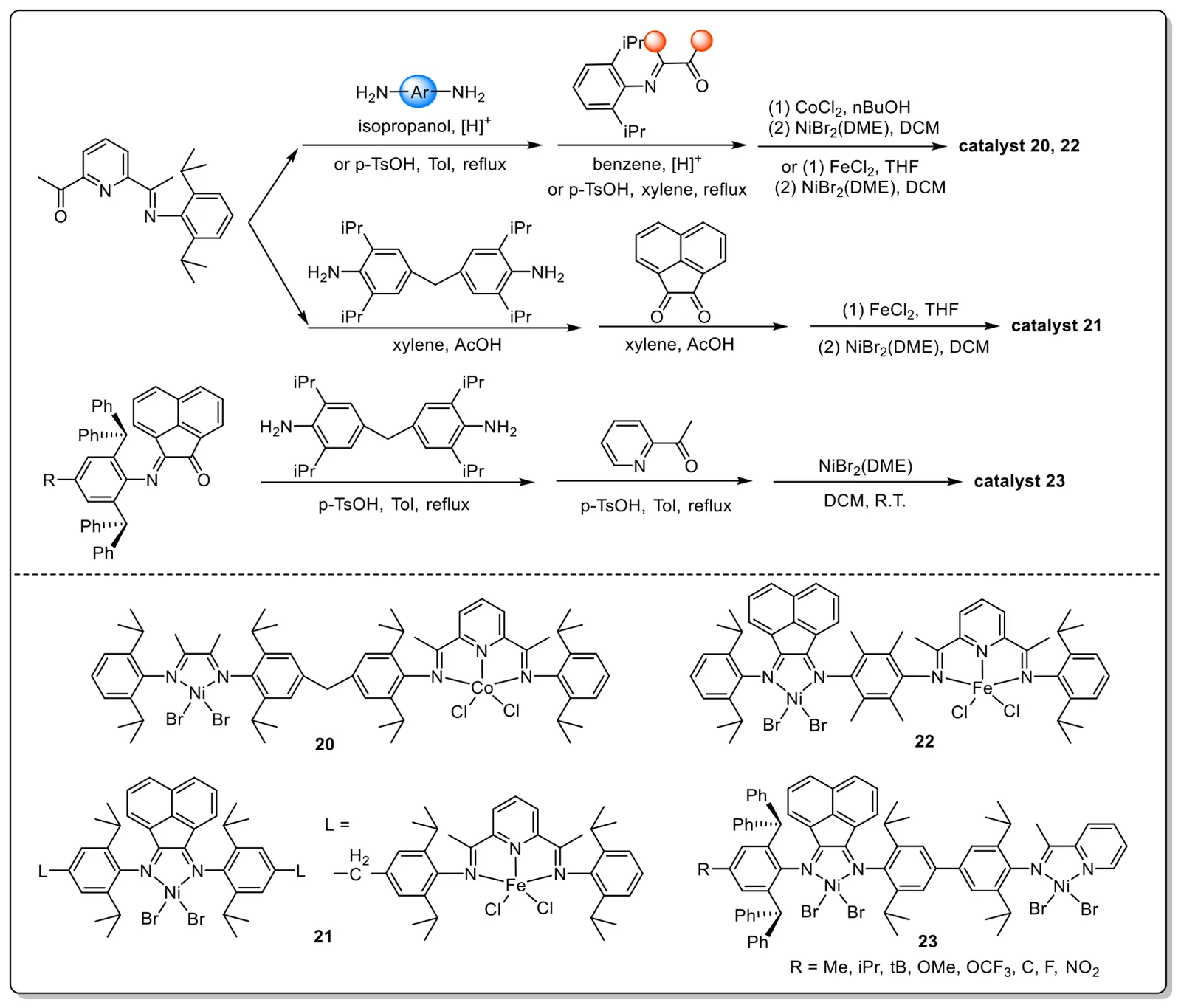
Synthesis and structural scheme of the special unsymmetric homo/hetero-binuclear α-dimine nickel catalysts.

Wang et al. [78] prepared a heterobinuclear Ni-Co complex incorporating both α-diimine and pyridine diimine units (Figure 6 (**20**)) in ethylene polymerization. When triethylaluminum (TEA) or MMAO was used as a cocatalyst, the ethylene polymerization activity of catalyst **20** was lower than that of an equivalent mixture of the mononuclear complexes Ni and Co. Furthermore, the nickel center in the binuclear complex **20** produced less high-molecular-weight polyethylene compared to the binary system. However, when TEA combined with [PhMe_2_NH][B(C_6_F_6_)_4_] was employed as the cocatalyst, the productivity of the nickel site improved, although the resulting polyethylene exhibited a lower degree of branching than that produced by the mononuclear Ni complex. UV-vis spectroscopy revealed that the absorption band corresponding to the active nickel center in catalyst **20** was weaker than that in the binary system. These results indicate that the productivity of the nickel center in the heterobinuclear complex is predominantly suppressed due to selective activation of the metal centers.

Based on a bimetallic heterocatalyst precursor, Kim et al. [79] successfully synthesized a novel trinuclear Fe(II)/Ni(II) heteronuclear catalyst for ethylene polymerization, which contains two Fe centers and one Ni center (Figure 6 (**21**)). When activated with MAO or common alkyl aluminums such as Et_2_AlCl and TMA, all the catalysts exhibited ethylene polymerization activities exceeding 10^5^ g(PE)/(mol(Metal)·h) at 30 °C. Compared with monometallic Ni analogs and homobimetallic Fe analogs, this heterotrimetallic catalyst **21** demonstrated higher catalytic activity and a longer life time, particularly under high temperature polymerization conditions.

Khoshsefat et al. [80] synthesized a heterodinuclear Fe-Ni catalyst **22** (Figure 6) and employed it in ethylene polymerization under Zn-assisted cooperative conditions to investigate the effect of adjacency of second metal center on chain transfer efficiency. Compared with mononuclear analogs and homobimetallic catalysts, this heterobinuclear catalyst **22** was capable of synergistically generating random copolymers containing both short-chain and long-chain branches in the presence of diethyl zinc. Moreover, under low and high diethyl zinc concentrations, block copolymers were obtained via coordination chain transfer polymerization (CCTP) and chain shuttling polymerization (CSP), respectively.

Recently, Sun et al. [81] integrated α-diimine units with pyridine–imine units into a single ligand, efficiently synthesizing a series of ligands featuring a para-biphenylene-linked structure through a two-step procedure. The electronic effects of these ligands can be systematically tuned by varying the para-substituents on the benzene rings. The reaction of these ligands with two equivalents of NiBr_2_(DME) resulted in the successful formation of binuclear nickel complexes (Figure 6 (**23**)). Following activation with AlMe_3_ or Me_2_AlCl, these catalysts demonstrated moderate to high catalytic activity in ethylene polymerization, with the most active catalyst exhibiting a activity of 8.5 × 10^6^ g(PE) mol(Ni)^−1^ h^−1^. The resulting polyethylene exhibited a discernible bimodal molecular weight distribution, with the ratio of the high-molecular-weight component to the low-molecular-weight component being significantly influenced by the polymerization temperature. It is noteworthy that the obtained polyethylenes all possessed highly branched structures. Consequently, these bimodal materials exhibited superior tensile strength and elastic recovery compared to the unimodal polyethylene prepared using mononuclear nickel catalysts. This finding demonstrates their potential application as thermoplastic elastomers.

## 6. Conclusions

Binuclear α-diimine nickel catalysts have shown significant promise in ethylene polymerization owing to their unique intermetallic cooperativity and structurally tunable designs. This review has systematically surveyed four catalyst classes including rigid aryl-bridged, flexible chain-bridged, N-heterocyclic-bridged, and specialty unsymmetric systems. The bridging unit critically governs intermetallic distance, orientation, and cooperative effects, thereby influencing catalytic activity, thermal stability, and chain-walking behavior. Through rational design, certain binuclear catalysts have achieved superior activity and unique microstructural control (branching type, density, and molecular weight distribution) compared to mononuclear analogs, enabling tailored synthesis of novel polyethylens.

The most prominent advantage of binuclear α-diimine nickel catalysts lies in their unique chain-walking mechanism, which enables flexible regulation of the microstructural control of the resulting polyethylene through straightforward modifications of the ligand structure. In addition, these catalysts exhibit the capability for copolymerization with comonomers, offering a promising route to overcome the longstanding challenge associated with conventional catalysts in preparing functionalized polyolefins. Nevertheless, several limitations remain to be addressed. Their thermal stability is still insufficient, as the polymerization temperature generally must be maintained below 90 °C; otherwise, a marked decline in activity or even complete deactivation occurs. Moreover, as homogeneous catalytic systems, they are difficult to apply directly in existing industrial plants, owing to issues related to catalyst separation and polymer morphology control, although progress has been achieved in immobilization studies. Furthermore, in comparison with well-established Ziegler–Natta and metallocene catalysts, their industrial application remains at an exploratory stage. Thus, there still exist a multitude of challenges that need to be tackled before further advancement can be achieved. The bimetallic synergy mechanism requires direct spectroscopic evidence and theoretical support via in situ characterization and computational chemistry. Catalyst design remains largely empirical, necessitating a predictive platform for structure–activity relationships. Precise synergistic control over branching distribution, copolymer sequence, and molecular weight distribution is still elusive. Finally, long-term stability and cost-effectiveness under industrial conditions require further validation.

## Data Availability

No new data were created or analyzed in this study. Data sharing is not applicable to this article.
